# Transcranial Direct Current Stimulation Combined With Repetitive Transcranial Magnetic Stimulation for Depression: A Randomized Clinical Trial

**DOI:** 10.1001/jamanetworkopen.2024.44306

**Published:** 2024-11-13

**Authors:** Dongsheng Zhou, Xingxing Li, Shuochi Wei, Chang Yu, Dongmei Wang, Yuchen Li, Jiaxin Li, Junyao Liu, Shen Li, Wenhao Zhuang, Yanli Li, Ruichenxi Luo, Zhiwang Liu, Jimeng Liu, Yongming Xu, Jialin Fan, Guidong Zhu, Weiqian Xu, Yiping Tang, Raymond Y. Cho, Thomas R. Kosten, Xiang-Yang Zhang

**Affiliations:** 1 Department of Psychiatry, Affiliated Kangning Hospital of Ningbo University (Ningbo Kangning Hospital), Ningbo Key Laboratory for Physical Diagnosis and Treatment of Mental and Psychological Disorders, Ningbo, Zhejiang, China; 2 CAS Key Laboratory of Mental Health, Institute of Psychology, Chinese Academy of Sciences (CAS), Beijing, China; 3 Department of Psychiatry, Lishui’s Second People’s Hospital, Lishui, Zhejiang, China; 4 Department of Psychiatry, Taizhou Second People’s Hospital, Taizhou, Zhejiang, China; 5 Department of Psychology, University of Chinese Academy of Sciences, Beijing, China; 6 Psychoneuromodulation Center, Tianjin Anding Hospital, Mental Health Center of Tianjin Medical University, Tianjin, China; 7 Department of Psychiatry and Behavioral Sciences and The Menninger Clinic, Baylor College of Medicine, Houston, Texas

## Abstract

**Importance:**

Repetitive transcranial magnetic stimulation (rTMS) and transcranial direct current stimulation (tDCS) are both recognized as effective treatments for depression when applied individually. However, it is unknown whether rTMS combined with tDCS has better efficacy in the treatment of major depressive disorder (MDD).

**Objective:**

To investigate the clinical effectiveness and safety of rTMS, tDCS, tDCS + rTMS, and sham tDCS + sham rTMS after 2 weeks of treatment in patients with MDD.

**Design, Setting, and Participants:**

This double-blind, sham-controlled randomized clinical trial was conducted from November 2021 to April 2023 at 3 hospitals in China (Kangning Hospital affiliated with Ningbo University, Lishui Second People’s Hospital, and Taizhou Second People’s Hospital). Adult patients (aged 18-65 years) who were diagnosed with major depressive disorder were recruited. Participants were randomly assigned to 1 of 4 interventions: active tDCS + active rTMS, sham tDCS + active rTMS, active tDCS + sham rTMS, and sham tDCS + sham rTMS. Data analysis followed an intention-to-treat approach.

**Intervention:**

Patients received a 2-week course of treatment. The tDCS was administered using a 2-mA direct current stimulator with electrodes placed on the left and right dorsolateral prefrontal cortex (DLPFC). Each tDCS session lasted 20 minutes and was conducted 30 to 60 minutes prior to the rTMS session for a total of 10 sessions. The rTMS was delivered at a frequency of 10 Hz using a figure-8 coil placed on the left DLPFC, with each session consisting of 1600 pulses. Treatments were administered 5 times per week for 2 weeks. Sham treatments were performed with a pseudostimulation coil and emitted only sound.

**Main Outcomes and Measures:**

The primary outcome was the change in total score from baseline to week 2 on the 24-item Hamilton Depression Rating Scale (HDRS-24; score range: 0-52, with the highest score indicating more severe symptoms).

**Results:**

A total of 240 participants (139 females [57.9%]; mean [SD] age, 32.50 [15.18] years) were included. As a primary outcome, patients who received active tDCS + active rTMS showed a significantly greater reduction in mean (SD) HDRS-24 total scores compared with patients in the other 3 groups (active tDCS + active rTMS: 18.33 [5.39], sham tDCS + active rTMS: 14.86 [5.59], active tDCS + sham rTMS: 9.21 [4.61], and sham tDCS + sham rTMS: 10.77 [5.67]; *F*_3,236_ = 35.79; η^2^ = 0.31 [95% CI, 0.21-0.39]; *P* < .001).

**Conclusions and Relevance:**

This trial found that tDCS + rTMS was a more effective and safe treatment option than either the tDCS or rTMS intervention alone for patients with MDD.

**Trial Registration:**

China Clinical Trial Registry Identifier ChiCTR2100052122.

## Key Points

**Questions:**

Does the combination of repetitive transcranial magnetic stimulation (rTMS) and transcranial direct current stimulation (tDCS) vs each treatment alone provide better outcomes in the treatment of depression?

**Findings:**

In this randomized clinical trial involving 240 participants, those who received active tDCS + active rTMS had a greater reduction in the 24-item Hamilton Depression Rating Scale total score than those who received sham tDCS + active rTMS, active tDCS + sham rTMS, or sham tDCS + sham rTMS after 2 weeks of treatment.

**Meaning:**

The findings indicate that tDCS + rTMS is more effective than either tDCS or rTMS alone in depression treatment and has a comparable safety profile.

## Teaser

This randomized clinical trial compares the outcomes of combining transcranial direct current stimulation (tDCS) with repetitive transcranial magnetic stimulation (rTMS) instead of applying them individually in patients with a major depressive disorder.

## Introduction

Given the high prevalence and substantial burden of major depressive disorder (MDD), including the risk of suicide, there is an urgent need for faster and more effective treatment modalities.^1,2^ Current treatments, such as selective serotonin reuptake inhibitors and serotonin-norepinephrine reuptake inhibitors, often exhibit a slow onset of action, are associated with adverse effects, and yield a substantial rate of nonresponse.^3^ In response to these limitations, noninvasive brain-stimulation techniques—namely, transcranial magnetic stimulation (TMS) and transcranial direct current stimulation (tDCS)—have emerged as promising alternative therapies.^4,5^ These modalities offer quicker alleviation of depressive symptoms with fewer adverse effects, compelling the exploration of their combined application for faster enhanced therapeutic outcomes.

Transcranial magnetic stimulation uses a transient magnetic field generated by a coil to penetrate the skull and stimulate neural tissue, thereby modulating neural circuits. The US Food and Drug Administration has approved repetitive TMS (rTMS), particularly high-frequency rTMS targeting the left dorsolateral prefrontal cortex (DLPFC), for treating MDD.^6,7,8^ A meta-analysis has shown that rTMS treatment directed at the left DLPFC is effective, with the observation that higher daily pulse and additional sessions can enhance efficacy.^9^ However, a challenge of rTMS is that its onset of action generally takes 4 to 6 weeks. In contrast, tDCS applies a weak direct current through scalp electrodes to modulate cortical excitability.^10,11^ A large study demonstrated for the first time that, although tDCS was not inferior to escitalopram, its efficacy was better than that of placebo.^12^ Although tDCS demonstrates efficacy and fewer adverse effects, requiring more treatment courses limits its application in treatment of acute MDD.^13,14^

The literature indicates that excitability of the cortex can be maintained for up to 90 minutes after a single use of tDCS.^15^ Further research indicates that prestimulating with tDCS to shift neuronal resting membrane potential, followed by rTMS to generate neuronal action potential, may induce more enduring changes in cortical excitability and plasticity. This potential synergistic effect between tDCS and rTMS is focused on shortening the onset of therapeutic efficacy.^16^ Conclusions are inconsistent about whether shorter intervention cycles can produce a favorable antidepressant effect.^6^ Therefore, we conducted a randomized clinical trial to explore whether the combination of tDCS and rTMS is more effective than a single treatment modality over 2 weeks and whether this dual treatment modality can provide similar rapid-acting outcomes in the treatment of depression.

Considering the effectiveness of tDCS and rTMS in mental disorders and their potential synergistic benefits for depression treatment, we aimed to investigate the effectiveness and safety of a 2-week course of rTMS, tDCS, tDCS + rTMS, and sham tDCS + sham rTMS in patients with MDD. We hypothesized that, after 10 sessions, tDCS + rTMS would exhibit the greatest score reduction in the 24-item Hamilton Depression Rating Scale (HDRS-24; score range: 0-52, with the highest score indicating more severe symptoms), thus offering a rapid-acting treatment alternative for depression. Additionally, we aimed to compare the adverse effects, response rates, and remission rates 2 weeks after completion of noninvasive brain stimulation, anticipating that the dual treatment modality could provide a therapeutic advantage.

## Methods

### Study Design

This double-blind, sham-controlled randomized clinical trial was conducted from November 2021 to April 2023 and consisted of 2 weeks of treatment and 2 weeks of follow-up. All participants underwent clinical symptom assessment at baseline, at the end of treatment (week 2), and at the end of follow-up (week 4). The detailed protocol is available in Supplement 1. The Ningbo Kangning Hospital Ethics Committee approved this study. All research procedures were conducted in accordance with the Declaration of Helsinki.^17^ All participants provided written informed consent. We followed the Consolidated Standards of Reporting Trials (CONSORT) reporting guideline.

Note: we conducted a continuation trial to assess a secondary outcome of cognitive dysfunction following an approved amendment to the trial protocol. For this secondary analysis, not all original trial participants completed baseline cognitive assessments. In total, 178 participants from the cognitive outcome analysis were also included in the depression outcome analysis of the primary trial reported herein. To ensure adequate baseline cognitive assessment for the continuation study, and following the approval of a protocol amendment, an additional 62 patients with major depressive disorder who completed baseline cognitive assessments were subsequently recruited for the secondary outcome analysis through October 2023, analyzed, and are reported elsewhere.^18^ The depression outcome analysis that is reported herein represents the primary report of this randomized clinical trial.

### Participants and Selection Criteria

We recruited 240 patients who met the inclusion criteria from a cohort of 550 patients screened at 3 hospitals in China (Kangning Hospital affiliated with Ningbo University, Lishui Second People’s Hospital, and Taizhou Second People’s Hospital). The inclusion criteria were (1) diagnosis of MDD, as defined in the *Diagnostic and Statistical Manual of Mental Disorders* (Fifth Edition), by 2 independent psychiatrists; (2) HDRS-24 score higher than 20; (3) aged 18 to 65 years with right-handedness; (4) ability to tolerate the treatment; (5) hospitalization prior to recruitment; and (6) agreement to participate in this study and sign a consent form.

Exclusion criteria were (1) history of epilepsy, brain tumor, or trauma; (2) history of TMS, tDCS, or electroconvulsive therapy within the past 3 months; and (3) presence of metal implants. Criteria for loss or withdrawal were (1) refusal of treatment on 2 or more occasions; (2) serious adverse effects and inability to tolerate treatment; (3) sudden deterioration of the condition during the study period requiring a change of medication or other treatment; and (4) changes in medication type and dose during hospitalization and during follow-up.

### Randomization, Treatment, and Blinding

**Figure 1.**
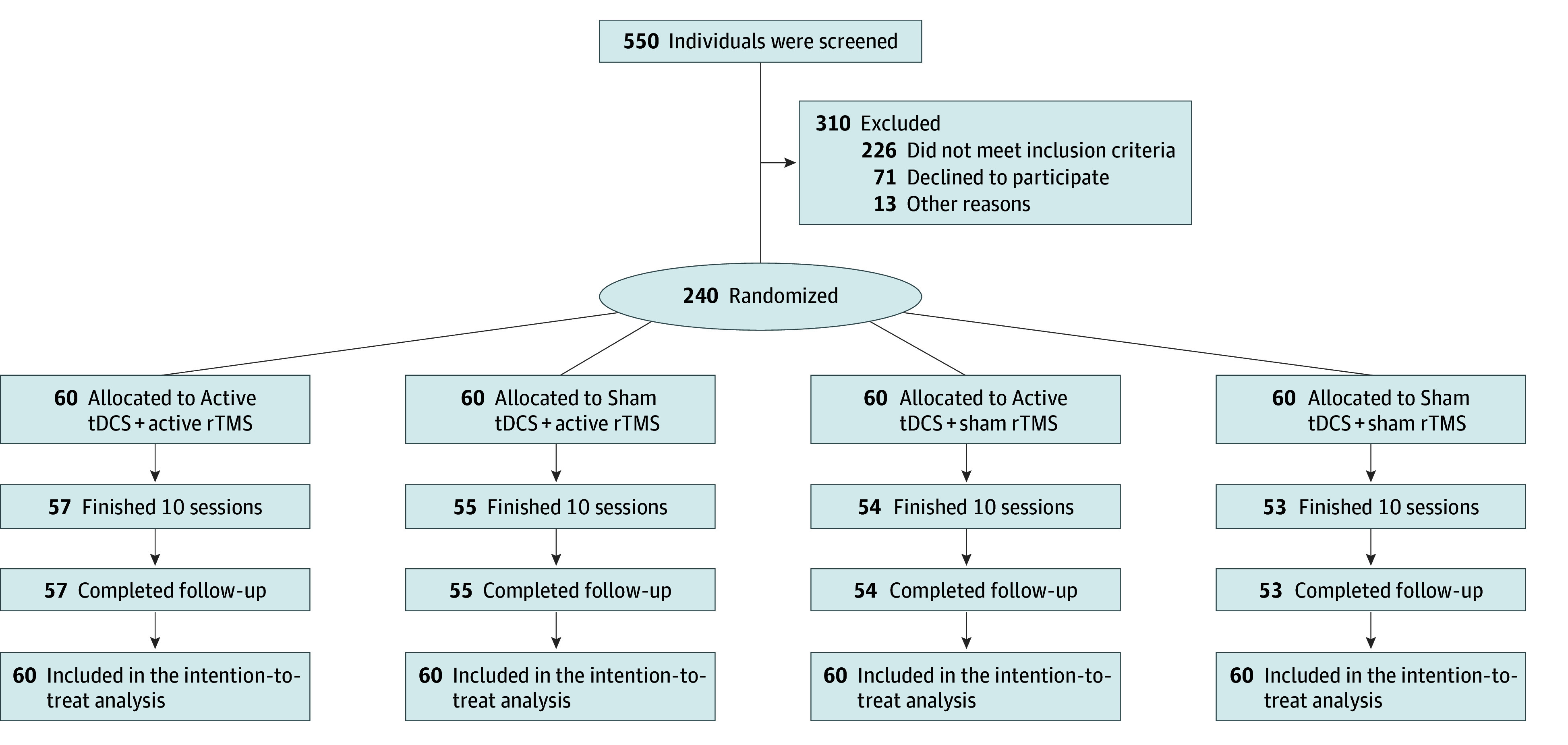
Trial Flow Diagram rTMS indicates repetitive transcranial magnetic stimulation; tDCS, transcranial direct current stimulation.

The flow diagram for this trial is shown in Figure 1. We used a computer-generated random number table to create the randomization sequence for assigning all patients into 4 groups (active tDCS + active rTMS, sham tDCS + active rTMS, active tDCS + sham rTMS, and sham tDCS + sham rTMS) with a 1:1:1:1 ratio, ensuring that each participant had an equal chance of being assigned to any group. To prevent selection bias, allocation concealment was implemented using sealed, opaque envelopes. Throughout the study, both participants and the researchers directly involved in treatment and assessment were kept masked, and 2 unblinding tests were conducted at the end of the trial (eMethods in Supplement 2).

### Targeting and Procedure

Before targeted therapy, magnetic resonance imaging (MRI) was used to obtain 3-dimensional T1-weighted images, which were imported into the Brainsight TMS navigation system (Rogue Research Inc). Left and right DLPFC targets were set at Montreal Neurological Institute coordinates (−44, 40, 29) and (44, 40, 29).^19^ To ensure accuracy, navigation was used throughout the rTMS treatment. For tDCS treatment, navigation was used only prior to the first treatment to pinpoint the target area.

The tDCS stimulators (Foc.us Ltd) delivered 2-mA direct current through 5 × 5 cm^2^ sponge electrodes placed with the anode on the left DLPFC and cathode on the right DLPFC. Each tDCS session lasted 20 minutes at a fixed time of day and was performed approximately 30 to 60 minutes before rTMS from Monday to Friday, with a weekend break, for a total of 10 treatments over 2 weeks. The same treatment protocol was used for the active or sham tDCS group. For the sham tDCS group, patients felt current stimulation for 30 seconds and then the current was gradually decreased to 0 mA.

We used a Magstim Rapid stimulator (Magstim Ltd) for the rTMS treatment, with all patients receiving 10-Hz stimulation via a figure-8 coil on the left DLPFC. Each session included 40 trains (1600 pulses) with 4-second intervals and 26-second intertrain breaks. Treatments were delivered at 110% resting motor threshold from Monday through Friday for 2 weeks.

Sham stimulation treatments were performed using a pseudostimulation coil (Coil-D70air film; Magstim Ltd). This coil was placed on the left DLPFC and emitted only sound without stimulation.

### Clinical Assessment and Outcomes

Efficacy and adverse events were assessed at baseline, at the end of 2-week treatment, and during the 2-week follow-up period. The primary outcome was the change in HDRS-24 total score from baseline to week 2. The secondary outcomes included (1) the change in HDRS-24 total score from baseline to week 4; (2) remission rate, defined as the HDRS-24 total score of 9 or lower, at weeks 2 and 4; (3) response rate, defined as 50% or greater reduction in HDRS-24 total score, from baseline to week 2 and week 4; and (4) adverse events.

All HDRS-24 evaluations were conducted by neuropsychologists blinded to participants’ interventions. These neuropsychologists underwent specialized training in HDRS-24 assessment before the study. An interrater correlation coefficient of at least 0.8 was required to ensure consistency. If the interrater correlation coefficient fell below this threshold, the discrepancies were addressed and 2 additional patient assessments were conducted to validate improved consistency. This process ensured high interrater agreement and reliability in HDRS-24 scores, maintaining assessment accuracy throughout the study.

### Sample Size Calculation

We calculated the sample size required to adequately estimate the change in the RBANS (Repeatable Battery for the Assessment of Neuropsychological Status) total score difference between the 4 groups at the medium effect size (0.25), power of 95%, a 2-tailed α level (5%), an *F* test, repeated measures multivariate analysis of variance, and between-factor model. The correlation between repeated measures was 0.5. The minimum total sample size was 188.

### Statistical Analysis

All statistical analyses were performed using SPSS 22.0 (IBM). Normality was assessed with the Kolmogorov-Smirnov test, and sphericity and homogeneity of variance were tested with Mauchly and Levene tests. Analysis of variance and χ^2^ tests were used to assess differences in demographic and clinical variables between groups. Intention-to-treat analysis was conducted, with missing data estimated by mean interpolation. Multivariate analysis of variance was used to analyze changes in HDRS-24 scores across 3 time points (baseline, week 2, and week 4) and 4 intervention groups (active tDCS + active rTMS, sham tDCS + active rTMS, active tDCS + sham rTMS, and sham tDCS + sham rTMS). If significant, analysis of covariance was applied. Bonferroni correction controlled for multiple testing.

We calculated changes in HDRS-24 total score for the 4 groups (week 2 and week 4 minus baseline), and then we used analysis of variance to compare the mean reduction in HDRS-24 total score between the 4 groups. Clinical remission (HDRS-24 score <9) and response (HDRS-24 score decrease ≥50%) were compared using the χ^2^ test. Statistical significance was set at *P* < .05, and all tests were 2-tailed.

## Results

### Demographic and Basic Descriptive Data

A total of 240 eligible patients were recruited and randomized to the 4 intervention groups (Figure 1). Participants included 139 females (57.9%) and 101 males (42.1%), with a mean (SD) age of 32.50 (15.18) years. There were no significant differences in the demographic and clinical characteristics as well as antidepressant type at baseline between the 4 groups (Table 1). During the course of treatment, 219 patients (91.3%) completed both a 2-week intervention and assessment and a 2-week follow-up. A total of 21 patients dropped out during the study. Most patients in each group (eg, 52 of 55 [94.6%] who received sham tDCS + active rTMS) believed they received the actual treatment, and the raters were unable to accurately identify the specific treatment they received (eResults in Supplement 2).

**Table 1.** Demographic and Clinical Characteristics of Patients by Intervention Group

Characteristic	Patients, mean (SD)			
	Active tDCS + active rTMS (n = 60)	Sham tDCS + active rTMS (n = 60)	Active tDCS + sham rTMS (n = 60)	Sham tDCS + sham rTMS (n = 60)
Age, y	33.62 (15.32)	31.98 (14.46)	31.67 (15.69)	32.73 (15.51)
Sex, No. (%)				
Female	38 (63.3)	34 (56.7)	36 (60.0)	31 (51.7)
Male	22 (36.7)	26 (43.3)	24 (40.0)	29 (48.3)
Disease duration, y	4.55 (5.03)	3.40 (3.55)	5.50 (6.22)	4.92 (5.60)
Educational level, y	10.15 (3.38)	11.00 (3.40)	10.93 (3.13)	9.98 (3.24)
Suicidal ideation (yes)	23 (38.33)	25 (41.67)	22 (36.67)	25(41.67)
Antidepressants type, No. (%)				
Escitalopram	30 (50.0)	30 (50.0)	29 (48.3)	34 (56.7)
Fluoxetine	7 (11.7)	8 (13.3)	10 (16.7)	5 (8.3)
Sertraline	11 (18.3)	7 (11.7)	7 (11.7)	6 (10.0)
Venlafaxine	7 (11.7)	7 (11.7)	6 (10.0)	8 (13.3)
Duloxetine	5 (8.3)	8 (13.3)	8 (13.3)	7 (11.7)
HDRS-24 score	26.37 (5.62)	25.98 (4.50)	24.92 (4.09)	25.87 (5.43)

Abbreviations: rTMS, repetitive transcranial magnetic stimulation; tDCS, transcranial direct current stimulation; HDRS-24, 24-item Hamilton Depression Rating Scale (score range: 0-52, with the highest score indicating more severe symptoms).

### Primary Outcome

The HDRS-24 total score showed a significant group by time interaction (*F*_6,236_ = 20.70; η2 = 0.21; *P* < .001), a significant time effect (*F*_2,236_ = 1555.41; η2 = 0.87; *P* < .001), and a significant group effect (*F*_3,236_ = 10.22; η2 = 0.12; *P* < .001) (Table 2). The mean (SD) HDRS-24 total scores were 26.37 (5.62) at baseline and 8.04 (3.64) after treatment for active tDCS + active rTMS, 25.98 (4.50) at baseline and 11.13 (5.83) after treatment for sham tDCS + active rTMS, 24.92 (4.09) at baseline and 15.70 (5.76) after treatment for active tDCS + sham rTMS, and 25.87 (5.43) at baseline and 15.09 (5.82) after treatment for sham tDCS + sham rTMS.

**Table 2.** 24-Item Hamilton Depression Rating Scale Scores at Baseline, Week 2, and Week 4 by Intervention Group

Group	No. of patients	HDRS-24 score, mean (SD)			Group effect, *F*^a^	Time effect, *F*^a^	Group by time interaction, *F*^a^
		Baseline	Week 2	Week 4			
Active tDCS + active rTMS	60	26.37 (5.62)	8.04 (3.64)	6.33 (3.25)	10.22	1555.41	20.70
Sham tDCS + active rTMS	60	25.98 (4.50)	11.13 (5.83)	8.64 (3.02)			
Active tDCS + sham rTMS	60	24.92 (4.09)	15.70 (5.76)	8.20 (2.71)			
Sham tDCS + sham rTMS	60	25.87 (5.43)	15.09 (5.82)	9.28 (1.96)			

Abbreviations: rTMS, repetitive transcranial magnetic stimulation; tDCS, transcranial direct current stimulation; HDRS-24, 24-item Hamilton Depression Rating Scale.

^a^ *P* < .001.

**Figure 2.**
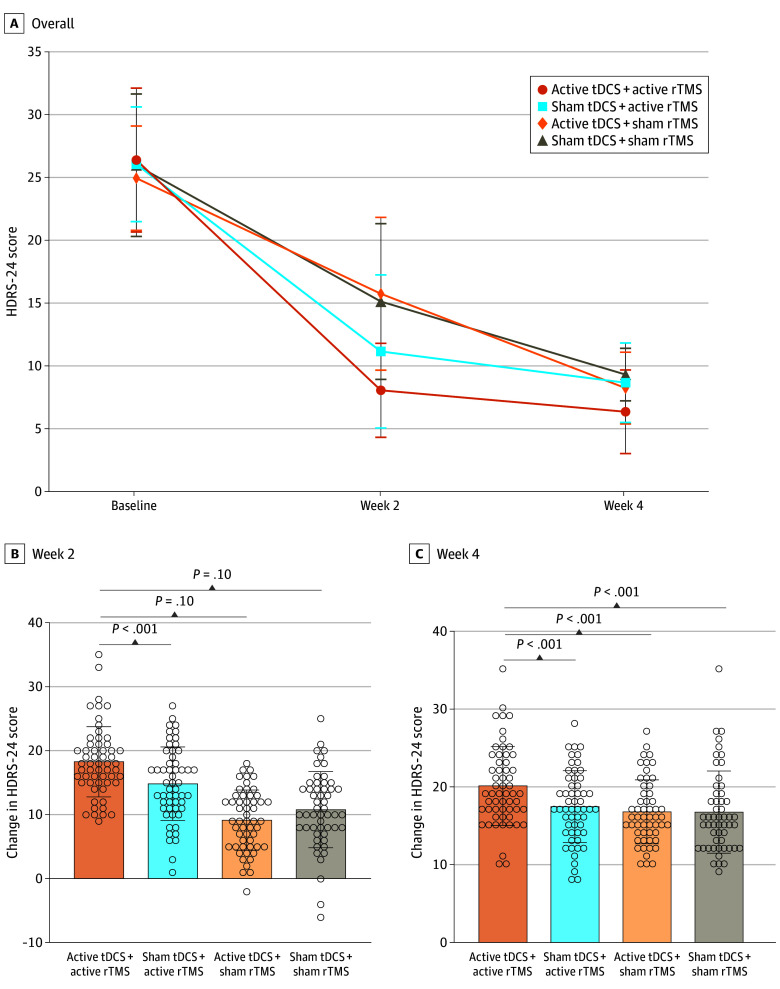
Hamilton Depression Rating Scale (HDRS)-24 Scores for Each Intervention Group at Baseline, Week 2, and Week 4 The HDRS-24 score range was 0 to 52, with the highest score indicating more severe symptoms. rTMS indicates repetitive transcranial magnetic stimulation; tDCS, transcranial direct current stimulation. Error bars represent 95% CIs. Circles indicate patient scores.

There was also a statistically significant difference in the reduction of mean (SD) HDRS-24 total score across the 4 groups (active tDCS + active rTMS: 18.33 [5.39], sham tDCS + active rTMS: 14.86 [5.59], active tDCS + sham rTMS: 9.21 [4.61], and sham tDCS + sham rTMS: 10.77 [5.67]; *F*_3,236_ = 35.79; η^2^ = 0.31 [95% CI, 0.21-0.39]; *P* < .001) (Table 3). Post hoc tests showed that compared with the other 3 groups, the active tDCS + active rTMS group displayed the greatest reduction in HDRS-24 total score (vs sham tDCS + active rTMS: η^2^ = 0.09 [95% CI, 0.02-0.20], *P* < .001; vs active tDCS + sham rTMS: η^2^ = 4.46 [95% CI, 0.33-0.56], *P* < .001; vs sham tDCS + sham rTMS: η^2^ = 0.32 [95% CI, 0.19-0.44], *P* < .001). We also found that those who received sham tDCS + active rTMS exhibited a significant reduction in HDRS-24 total score compared with the recipients of both active tDCS + sham rTMS (η^2^ = 0.24 [95% CI, 0.11-0.36]; *P* < .001) and sham tDCS + sham rTMS (η^2^ = 0.12 [95% CI, 0.03-0.23]; *P* < .001). We did not find any significant difference between active tDCS + sham rTMS and sham tDCS + sham rTMS (η^2^ = 0.02 [95% CI, 0.001-0.01]; *P* = .10) (Figure 2).

**Table 3.** Primary and Secondary Indicators by Intervention Group

Indicator	Active tDCS + active rTMS (n = 60)	Sham tDCS + active rTMS (n = 60)	Active tDCS + sham rTMS (n = 60)	Sham tDCS + sham rTMS (n = 60)
Primary outcome				
Decrease in HDRS-24 score, baseline to week 2, mean (SD)	18.33 (5.39)	14.86 (5.59)	9.21 (4.61)	10.77 (5.67)
Secondary outcomes				
Decrease in HDRS-24 score, baseline to week 4, mean (SD)	20.03 (4.97)	17.35 (4.52)	16.71 (4.03)	16.58 (5.07)
Response 1 at week 2, No. (%)	51 (85.0)	44 (73.3)	18 (30.0)	19 (31.7)
Remission 1 at week 2, No. (%)	30 (50.0)	28 (46.7)	15 (25.0)	8 (13.3)
Response 2 at week 4, No. (%)	55 (91.7)	53 (88.3)	54 (90.0)	55 (91.7)
Remission 2 at week 4, No. (%)	50 (83.3)	37 (61.7)	43 (71.7)	33 (55.0)

Abbreviations: rTMS, repetitive transcranial magnetic stimulation; tDCS, transcranial direct current stimulation; HDRS-24, 24-item Hamilton Depression Rating Scale.

### Secondary Outcomes

At follow-up, HDRS-24 total score reductions significantly differed among the 4 intervention groups (η^2^ = 0.08; 95% CI, 0.02-0.15), with post hoc analyses showing the active tDCS + active rTMS group had greater reductions than the other groups (vs sham tDCS + active rTMS: η^2^ = 0.08 [95% CI, 0.01-0.18]; vs active tDCS + sham rTMS: η^2^ = 0.12 [95% CI, 0.03-0.23]; vs sham tDCS + sham rTMS: η^2^ = 0.11 [95% CI, 0.03-0.22]). After 2 weeks, recipients of active tDCS + active rTMS had a higher response rate than active tDCS + sham rTMS (51 [85.0%] vs 18 [30.0%]) and sham tDCS + sham rTMS (51 [85.0%] vs 19 [31.7%]). Additionally, recipients of active tDCS + active rTMS had a higher remission rate than those assigned to other groups after 2 weeks (30 [50.0%] vs 28 [46.7%] for sham tDCS + active rTMS, 15 [25.0%] for active tDCS + sham rTMS, and 8 [13.33%] for sham tDCS + sham rTMS). At week 4, response rates were similar across groups, but recipients of active tDCS + active rTMS showed a significantly higher remission rate (50 [83.3%]) than recipients of other interventions (*P* < .001) (Table 3). We also calculated the odds ratios (ORs) and risk differences (RDs) for each pair of groups at week 2 and week 4 (eTable 1 in Supplement 2). For example, comparing active tDCS + active rTMS to sham tDCS + sham rTMS, the OR for remission at 2 weeks was 6.50 (95% CI, 2.64-15.99) and the RD was 0.37 (95% CI, 0.21-0.52), suggesting that active tDCS + active rTMS had a higher remission than sham tDCS + sham rTMS at this time point.

### Adverse Events and Safety

During treatment and follow-up, no serious adverse events occurred. Adverse effects included skin redness (7 [2.9%]), dizziness (2 [0.8%]), headaches (8 [3.3%]), insomnia (4 [1.7%]), nausea (1 [0.4%]), mild irritation (5 [2.1%]), and pruritus (11 [4.6%]). All patients tolerated the treatments well, with no seizures or manic symptoms (eTable 2 in Supplement 2).

## Discussion

To our knowledge, this trial was the first to evaluate the safety, feasibility, and efficacy of combining tDCS and rTMS in treating depression. The major findings were as follows: (1) active tDCS + active rTMS had the highest reduced HDRS-24 total scores both at the postintervention and follow-up periods; (2) at week 2, sham tDCS + active rTMS showed significantly reduced HDRS-24 total scores compared with active tDCS + sham rTMS and sham tDCS + sham rTMS; (3) active tDCS + active rTMS had higher response rates at week 2 and higher remission rates at follow-up than other interventions; and (4) no serious adverse effects were observed in all 4 groups, and all patients tolerated the treatments well. These results suggest that tDCS + rTMS had a relatively better effect on depressive symptoms than other treatments.

In the 2-week trial, patients who received active tDCS + active rTMS treatment experienced significantly decreased HDRS-24 total scores than the comparison groups. This effect not only persisted throughout the 2-week follow-up but also was more beneficial than active rTMS alone. To our knowledge, this study is the first time that this combination of tDCS and rTMS has been applied in the treatment of depression. Some previous studies found that the dual brain stimulations might have benefits for other neurological disorders. For instance, anodal tDCS targeting the left primary motor cortex alongside high-frequency rTMS on the right primary motor cortex has been shown to induce substantial interhemispheric modulation and plasticity, improving cortical excitability and motor functions in healthy individuals.^20^ Similar synergy in combined tDCS and high-frequency rTMS applications yielded superior motor performance in stroke rehabilitation, surpassing the outcomes achieved with high-frequency rTMS alone.^21,22^ The success of this clinical trial not only underscores the potential of tDCS + rTMS therapy in effectively reducing depressive symptoms but also aligns with a growing body of research emphasizing the advantages of combined treatment approaches.^23,24,25^

Both tDCS and rTMS were effective at improving depressive symptoms. The potential mechanism of the combination might be a preconditioning effect of tDCS, which by depolarizing neurons and enhancing cortical excitability, would make subsequent rTMS more effective in generating action potential. This sequence of stimulation, starting with tDCS and then followed by high-frequency rTMS targeted at the left DLPFC, facilitates more profound and enduring modifications in cortical excitability and brain plasticity. Such changes are critical for achieving long-term potentiation within neurons, an indicator of effective depression treatment.^16^ However, recent computational modeling studies of tDCS on the left or right DLPFC have shown that the frontopolar area is stimulated by a stronger electric field than the region directly below the anode electrode (ie, left DLPFC).^26^ This finding suggests that although the anode is located on the left DLPFC and the cathode is located on the right DLPFC, the stimulation may actually enhance the medial frontal brain region. These findings are consistent with previous MRI-based electric field modeling results, which showed electric field strength in the frontopolar area.^27^ Additionally, prefrontal tDCS improved early surgical skill acquisition, and different electrophysiological responses were observed in patients with depression and schizophrenia, suggesting that combining tDCS and rTMS may have potential benefits for treating these conditions.^28,29^

### Limitations

This study has several limitations. First, the brief duration encompassing 10 treatment sessions may not suffice for tDCS and rTMS to manifest their full antidepressant potential, suggesting a need for extended treatment periods. Second, we were unable to regulate the medication regimens of participants given that all participants were on antidepressant medications throughout the study, which is reflective of clinical scenarios. Third, we did not perform stratified randomization or center-effects adjustment, which may introduce variability. Future studies should incorporate these considerations to enhance the robustness of the findings. In addition, future studies should incorporate multidisciplinary approaches, including electrophysiological, MRI, and biomarker analyses, to elucidate the mechanisms behind the therapeutic effects of combined tDCS and rTMS treatment.

## Conclusions

In this trial, we found that active tDCS + active rTMS was an effective and safe treatment option for patients with MDD. Future studies should focus on investigating the mechanism of this synergistic effect and improving the stimulation parameters to optimize the therapeutic effect.

## Notes

Supplement 1.
Trial Protocol

Supplement 2. **eMethods.** Randomization, rTMS or tDCS Treatment and Blinding **eResults.** Dropout and Integrity of Blinding **eTable 1.** Odds Ratios (OR) and Risk Difference (RD) of Remission and Response Rates Between Groups **eTable 2.** Adverse Events Among Patients Receiving Treatment

Supplement 3.
Data Sharing Statement
