# Omitted Mention of and Reference to Related Study

**DOI:** 10.1001/jamanetworkopen.2026.6508

**Published:** 2026-03-13

In the initial publication of the Original Investigation titled “Transcranial Direct Current Stimulation Combined With Repetitive Transcranial Magnetic Stimulation for Depression: A Randomized Clinical Trial,”^1^ published November 13, 2024, the authors omitted mention of a related study published in *BMC Medicine*^2^ that included most of the same participants. The article published in *JAMA Network Open*^1^ is the primary analysis of the randomized clinical trial, while that published in *BMC Medicine*^2^ is a secondary analysis. A paragraph has been added to the Methods section of the article published in *JAMA Network Open*^1^ explaining the distinction between the 2 articles. The article has been corrected.^1^
