# Supplementary material for: Transcranial Direct Current Stimulation Combined With Repetitive Transcranial Magnetic Stimulation for Depression: A Randomized Clinical Trial
Source: JAMA Netw Open. 2024 Nov 13;7(11):e2444306. doi: 10.1001/jamanetworkopen.2024.44306 (PMC11561687; doi:10.1001/jamanetworkopen.2024.44306)
Supplement: Supplement 1. — Trial Protocol [file jamanetwopen-e2444306-s001.pdf]

Final Research Protocol

Title of study:

Efficacy and acceptability of rTMS combined with tDCS for depression: A randomized clinical trial

Research team:

Dongsheng Zhou [1, #], Xingxing Li [1, 2, 5 #], Shuochi Wei [1, #], Chang Yu [1], Dongmei Wang [2, 5,] Yuchen Li [1], Jiaxin Li [2, 5], Junyao Liu [2, 5], Shen Li [6], Wenhao Zhuang [1], Yanli Li [1], Ruichenxi Luo [1], Zhiwang Liu [1], Jimeng Liu [1], Yongming Xu [1], Jialin Fan [3], Guidong Zhu [3], Weiqian Xu [4], Yiping Tang [4], Raymond Y. Cho [7], Thomas R. Kosten [7], Xiangyang Zhang [2, 5, *]

1 Department of psychiatry, Affiliated Kangning Hospital of Ningbo University（Ningbo Kangning Hospital）, Ningbo Key Laboratory for Physical Diagnosis and Treatment of Mental and Psychological Disorders, Ningbo, Zhejiang, China
2 CAS Key Laboratory of Mental Health, Institute of Psychology, Chinese Academy of Sciences, Beijing, China
3 The Second People's Hospital of Lishui, Lishui, Zhejiang, China.
4 Taizhou Second People's Hospital, Taizhou, Zhejiang, China.
5 Department of Psychology, University of Chinese Academy of Sciences, Beijing, China.
6 Psychoneuromodulation Center, Tianjin Anding Hospital, Mental Health Center of Tianjin Medical University, Tianjin, China
7 Department of Psychiatry and Behavioral Sciences, Baylor College of Medicine, and Menninger Clinic, Houston, TX, United States

# Dong-Sheng Zhou, Xingxing Li and Shuochi Wei contributed equally to this work and should be considered co-first authors.

*** Corresponding Authors:**

Dr. Xiang-yang Zhang; Institute of Psychology, Chinese Academy of Sciences, 16 Lincui Road, Beijing, 100101, China. Email: zhangxy@psych.ac.cn

Contents of the trial protocol

1 Synopsis of the study 1

2 Background and rationale 2

3 Hypotheses 3

4 Study design 3

5 Data analysis 7

6 Ethical considerations 7

7 Reference 10

1 Synopsis of the study

Study design	A randomized, double-blind, sham-controlled study
Study setting	Kangning Hospital affiliated with Ningbo University, Zhejiang, China<br>The Second People’s Hospital of Lishui, Zhejiang, China<br>Taizhou Second People's Hospital, Zhejiang, China
Study participants	Patients diagnosed as major depressive disorder
Primary measures	24-item Hamilton Depression Rating Scale (HDRS-24)
Primary objective	To evaluate the efficacy and acceptability of rTMS combined with tDCS for depression treatment
Primary outcomes	(1) Change in HDRS-24 in week 2
Randomization and interventions	Patients were randomly assigned to four groups (Group A: active rTMS + active tDCS, Group B: active rTMS +sham tDCS, Group C: sham rTMS + active tDCS, Group D: sham rTMS +sham tDCS)
Planned sample size	240 patients are required
Data analysis method	(1) Demographic and clinical variables between groups were analyzed using ANOVA for continuous variables and chi-square tests for categorical variables.<br>(2) Repeated measures (RM) multivariate analysis (MANOVA) was applied to analyze the change in HAMD scores from baseline to week four, with three main time points as within-effect repeated measures and four different intervention groups as between-effect repeated measures.

	(3) Differences in the proportion of patients with clinical remission and response in each group were compared using the χ2 test.
Trial registration no.:	ChiCTR2100052122

2 Background and rationale

Major depressive disorder (MDD) is a severe mental illness with a high prevalence leading to a substantial burden and suicide rate [1], and the standard SSRI and SNRI antidepressants take 4 to 6 weeks to relieve depression, but often with the cost of intolerable side effects [2], and a nearly 33% rate of non-response [3, 4]. As an alternative approach in recent years, non-invasive brain stimulation (NIBS) has effectively reduced depressive symptoms by directly modulating cortical activity with comparable efficacy and fewer side effects [5, 6].

The two most common types of NIBS are transcranial magnetic stimulation (TMS) and transcranial direct current stimulation (tDCS). Repetitive TMS (rTMS) is approved by the U.S. Food and Drug Administration (FDA) for the treatment of MDD [7]. High-frequency rTMS (HF-rTMS) targets the left dorsolateral prefrontal cortex (DLPFC) and modulates the functional connection of brain cortex to improve depressive symptoms [8-10]. Some studies have shown that tDCS reduces depressive symptoms compared to sham stimulation by modulating cortical excitability and altering the resting potential of the neural membrane [11, 12 13].

At a time when more rapid and acutely effective antidepressants such as ketamine and various NMDA antagonists are being discovered and implemented, it is worthy to study how quickly can NIBS induce a more rapid resolution of depressive symptoms by combining the rTMS and tDCS. This combination has its theoretical basement, that is tDCS can shift neuronal resting membrane potentials and rTMS can generate

neuronal action potentials, and such combination can lead to a more extensive and long-lasting neural changes, which might to be more effective in depression treatment.

Accordingly, this study aims to evaluate the efficacy and acceptability of rTMS combined with tDCS for depression treatment, using a randomized, double-blind, sham-controlled study.

3 Hypotheses

1) Patients in the rTMS combined with tDCS group would have the most change in HDRS-24 score after treatment.
2) Patients in the rTMS combined with tDCS group would have the same safty as the rTMS alone group.

4 Study design

4.1 Study participants

(1) Inclusion criteria:

- diagnosed with major depressive disorder according to DSM-V by two independent psychiatrists;
- HDRS-24 score more than 20;
- aged 18-65 years with right-handedness;
- able to tolerate the treatment;
- all the patients we recruited were hospitalized;
- agreeing to participate in this study and signed a consent form.

(2) Exclusion criteria:

- history of epilepsy, brain tumor, or trauma;
- history of TMS, tDCS, or ECT treatments within the past 3 months;
- presence of metal implants;
- acute or serious suicide ideation.

(3) Criteria for lose:

- refusals of treatment on two or more occasions;
- serious adverse effects and inability to tolerate treatment;
- sudden deterioration of the condition during the study period requiring a change of medication or other treatment;
- changes in medication type and dosage during hospitalization and during the follow-up stage.

4.4 Study procedure

(1) Trial visits

Figure 1 shows the flow chart of participants from screening to post-treatment. At baseline and 10 sessions after treatment the questionnaires were evaluated by specialized scale raters who were unaware of the patient's intervention. Study visits and assessments are presented in Fig. 2.

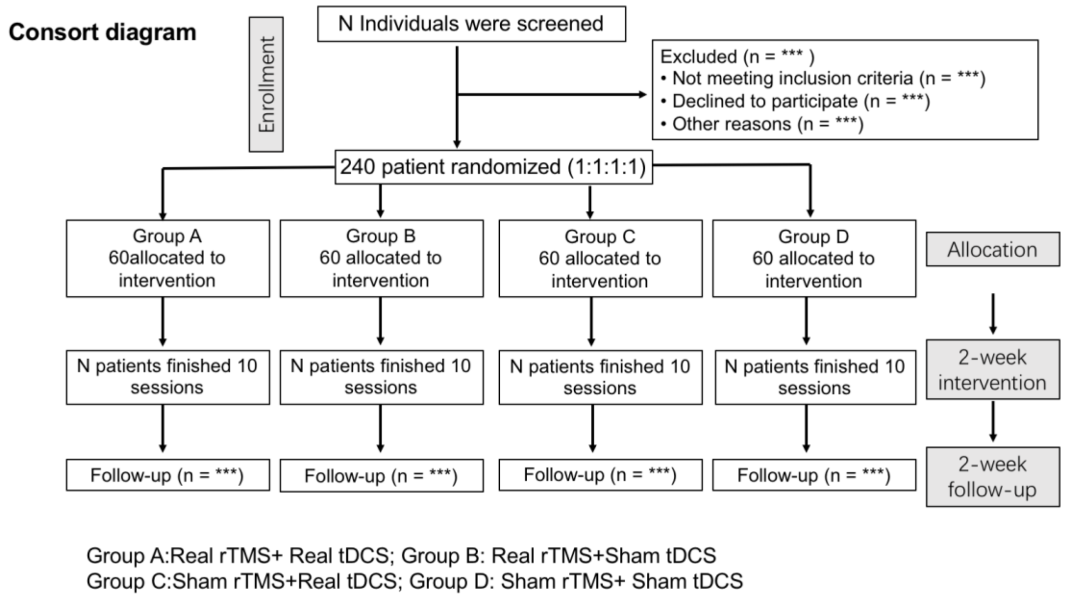

Figure 1. Flow chart of participants from screening to post-treatment.

	STUDY PERIOD							
	**Enrolment**	**Allocation**	**Post-allocation**				**Close-out**	**Follow-up**
**TIMEPOINT**		**0**	***Baseline***	***Day 1-5***	***Day 6-7***	***Day 8-12***	***Day 13***	***Day 26***
**ENROLMENT:**								
Eligibility screen	X							
Informed consent	X							
Allocation		X						
**INTERVENTIONS:**								
Group A				X		X		
Group B				X		X		
Group C				X		X		
Group D				X		X		
**ASSESSMENTS:**								
HDRS-24			X				X	X
Adverse events				X	X	X	X	X

Fig. 2 Standard protocol items: recommendations for Interventional Trials (SPIRIT) schedule for enrollment, treatment, and assessments.

(2) Protocol of rTMS

For rTMS treatment, the uniform MagStim Rapid stimulator (Magstim Ltd, Oxford, UK) for rTMS (active stimulation or sham stimulation) will be used on daily treatment, and patients will use the same machine throughout all interventions. Each patient will receive stimulation at 10 Hz using a figure-of-eight coil (Coil-D70-air membrane coil, Magstim) placed on the left DLPFC. The duration of the pulse sequence is 4-second

intervals with a 26-second inter-train interval. There is a total of 40 trains per session for a total of 1600 pulses. Treatments will occur Monday through Friday for 2 consecutive weeks at an intensity of 110% resting motor threshold (RMT). The sham treatment will be performed using a MAGSTIM pseudo-stimulation coil (Coil-D70air film, Magstim), placed on the left DLPFC, which do not transmit stimuli. The RMT is determined using an EMG instrument to observe the minimum stimulus intensity required to elicit a significant motor response in the right abductor pollicis brevis (APB) after a single stimulus (5 out of 10 times with an amplitude of 50 mV).

Stimulation of the left DLPFC is based on the Montreal Neurological Institute (MNI) coordinates of MNI x,y,z=44,40,29 and localized using the Brainsight neuronavigation system (Rogue Research Inc., Montreal, Canada).

(3) Protocol of tDCS

The tDCS stimulators supplied a battery-powered constant 2 mA DC stimulation (Foc.us Ltd., London, UK) through two 5x5 cm^2 sponge electrodes. The electrodes are wetted with normal saline during use, and trained nurses place the anode on the left DLPFC and the cathode on the right DLPFC. Each session last 20 minutes at a fixed time of day and is performed approximately 30 to 60 minutes before rTMS from Monday to Friday, with a weekend break, for a total of 10 treatments over 2 weeks. The same treatment protocol was used for the active or sham tDCS group. For the sham tDCS group, patients will feel current stimulation for 30s, and then the current gradually decrease to 0 ma.

(4) Protocol of the combination of rTMS and tDCS

All participants first will receive the standard tDCS protocol each day. At the end of tDCS treatment, approximately 30 to 60 minutes before rTMS protocol given the safety concerns of potentially inducing epilepsy.

4.5 Primary outcome measures

The primary outcome is the change in HDRS-24 total score from baseline to week 2. The secondary outcomes included: 1) follow up efficacy: defined as the change in HDRS-24 total score from baseline to week 4; 2) remission rate at weeks two and four: defined as the HDRS-24 total score ⩽ 9; 3) response rate, defined as ⩾ 50% reduction in HDRS -24 total score from baseline to week two and week four; 4) documented adverse events, including possible dizziness, burns, seizures, and headaches to assess the safety of the four interventions.

4.6 Sample size calculation

To detect the change of the difference between the four groups with enough statistical power, we estimated the sample size of patients with the effect size of 0.25, a power of 95% and a 2-tailed α level of 5%, selected the F tests, ANOVA: repeated measures, between factors model. The minimum total sample size required 188.

5 Data analysis

All statistical analyses were performed using SPSS 22.0 software. The Kolmogorov-Smirnov test was used to assess the normal distribution of continuous data, and the Mauchly ($p>0.05$) and Levene tests ($p>0.05$) were used to assess sphericity and homogeneity of variance, respectively. All data were normally distributed (Kolmogorov-Smirnov one-sample test: all $p>0.05$). Demographic and clinical variables between groups were analyzed using analysis of variance (ANOVA) for continuous variables and chi-square tests for categorical variables.

In this longitudinal study, the main aim was to examine the effects of four different intervention groups on depressive symptoms of MDD patients. Repeated measures (RM) multivariate analysis (MANOVA) was applied to analyze the change in HAMD scores from baseline to week four, with three main time points (baseline, week two,

week four) as within-effect repeated measures and four different intervention groups (A: active rTMS + active tDCS, B: active rTMS +sham tDCS, C: sham rTMS + active tDCS, D: sham rTMS +sham tDCS) as between-effect repeated measures. If the group × time interaction was significant, then the group difference at week 2 and 2-week follow-up was respectively analyzed by analysis of covariance (ANCOVA) with the baseline score as a covariate. If the interaction was not significant, no further statistical analyses were performed. To controlling for multiple testing, Bonferroni correction was applied.

We calculated changes in HDRS-24 total score for the four groups (week two and week four minus baseline, respectively), and then we used ANOVA to compare the mean reduction in HDRS-24 total score between the four groups. Patients were considered to be in clinical remission if the HDRS-24 total score decreased to less than 9 points, whereas patients were considered to have a clinical response if the HDRS-24 total score decreased by 50%. Differences in the proportion of patients with clinical remission and response in each group were compared using the χ^2 test.

6 Ethical considerations

6.1 Ethics approval and informed consent

Following and based on the Helsinki Declaration, this study was approved by the Ethics Committee of medical research in Ningbo Kangning Hospital. All participants provided signed informed consent before becoming involved in this study and could withdraw at any phase without giving a reason. The adverse events were continuously monitored; participants were asked to report any problems throughout their participation in the study. We also asked participants if they had any discomfort daily. An adverse event assessment was conducted to determine whether to terminate the study.

6.2 Data management

The research team ensured data confidentiality. The collected data was stored in the hard disk, which is protected by a password, and the research team was the only party able to access the database. The password was changed regularly. After the study, the hard disk is handed over to the person in charge of the experiment for safekeeping and if anyone needs it, they can apply for it.

7 Reference

1. Murray CJ, Barber RM, Foreman KJ, et al. Global, regional, and national disability-adjusted life years (DALYs) for 306 diseases and injuries and healthy life expectancy (HALE) for 188 countries, 1990-2013: quantifying the epidemiological transition. *Lancet.* 2015;386(10009):2145-91. https://doi.org/10.1016/s0140-6736(15)61340-x.
2. Santarsieri D, Schwartz TL. Antidepressant efficacy and side-effect burden: a quick guide for clinicians. *Drugs Context.* 2015;4:212290. https://doi.org/10.7573/dic.212290.
3. Rush AJ, Kraemer HC, Sackeim HA, et al. Report by the ACNP Task Force on response and remission in major depressive disorder. *Neuropsychopharmacology.* 2006;31(9):1841-53. https://doi.org/10.1038/sj.npp.1301131.
4. Cuijpers P, Karyotaki E, Weitz E, et al. The effects of psychotherapies for major depression in adults on remission, recovery and improvement: a meta-analysis. *J Affect Disord.* 2014;159:118-26. https://doi.org/10.1016/j.jad.2014.02.026.
5. Zewdie E, Ciechanski P, Kuo HC, et al. Safety and tolerability of transcranial magnetic and direct current stimulation in children: Prospective single center evidence from 3.5 million stimulations. *Brain Stimul.* 2020;13(3):565-575. https://doi.org/10.1016/j.brs.2019.12.025.
6. McClintock SM, Reti IM, Carpenter LL, et al. Consensus Recommendations for the Clinical Application of Repetitive Transcranial Magnetic Stimulation (rTMS) in the Treatment of Depression. *J Clin Psychiatry.* 2018;79(1). https://doi.org/10.4088/JCP.16cs10905.
7. George MS, Taylor JJ, Short EB. The expanding evidence base for rTMS treatment of depression. *Curr Opin Psychiatry.* 2013;26(1):13-8. https://doi.org/10.1097/YCO.0b013e32835ab46d.
8. Fox MD, Buckner RL, White MP, Greicius MD, Pascual-Leone A. Efficacy of transcranial magnetic stimulation targets for depression is related to intrinsic

functional connectivity with the subgenual cingulate. *Biol Psychiatry.* 2012;72(7):595-603. https://doi.org/10.1016/j.biopsych.2012.04.028.

9. Kito S, Hasegawa T, Takamiya A, et al. Transcranial Magnetic Stimulation Modulates Resting EEG Functional Connectivity Between the Left Dorsolateral Prefrontal Cortex and Limbic Regions in Medicated Patients With Treatment-Resistant Depression. *J Neuropsychiatry Clin Neurosci.* 2017;29(2):155-159. https://doi.org/10.1176/appi.neuropsych.15120419.

10. Padmanabhan JL, Cooke D, Joutsa J, et al. A Human Depression Circuit Derived From Focal Brain Lesions. *Biol Psychiatry.* 2019;86(10):749-758. https://doi.org/10.1016/j.biopsych.2019.07.023.

11. Brunoni AR, Moffa AH, Sampaio-Junior B, et al. Trial of Electrical Direct-Current Therapy versus Escitalopram for Depression. *N Engl J Med.* 2017;376(26):2523-2533. https://doi.org/10.1056/NEJMoa1612999.

12. Yokoi Y, Narita Z, Sumiyoshi T. Transcranial Direct Current Stimulation in Depression and Psychosis: A Systematic Review. *Clin EEG Neurosci.* 2018;49(2):93-102. https://doi.org/10.1177/1550059417732247.

13. Mutz J, Vipulananthan V, Carter B, et al. Comparative efficacy and acceptability of non-surgical brain stimulation for the acute treatment of major depressive episodes in adults: systematic review and network meta-analysis. *Bmj.* 2019;364:l1079. https://doi.org/10.1136/bmj.l1079.

14. Cristancho P, Cristancho MA, Baltuch GH, Thase ME, O'Reardon JP. Effectiveness and safety of vagus nerve stimulation for severe treatment-resistant major depression in clinical practice after FDA approval: outcomes at 1 year. *J Clin Psychiatry.* 2011;72(10):1376-82. https://doi.org/10.4088/JCP.09m05888blu.

Final Overview of Analyses

This study was designed to explore the clinical efficacy of rTMS combined with tDCS in the treatment of depression. To investigate whether rTMS combined with tDCS has an enhancing effect on antidepressant treatment of depression and is superior to single physiotherapy. To answer this question the study began with a randomized controlled trial protocol in which patients were randomized into four groups and given different physical therapy regimens, including rTMS + tDCS, rTMS + Sham tDCS, Sham rTMS+ tDCS, and Sham rTMS + Sham tDCS. Patients received two weeks of treatment for a total of 10 days and were assessed for depressive symptoms at the baseline period of enrollment and at the end of treatment, with follow-up at half a month.

We examined changes in depressive symptoms in the different physiotherapy regimen groups, with the aim of examining whether the rTMS + tDCS regimen had better changes in HDRS-24 score after treatment compared to rTMS alone, tDCS alone or both sham stimulations. Therefore, the statistical methods and ideas remained unchanged from the design's beginning until the experiment's end.

Statistical Plan for Main Outcome Paper

All statistical analyses were performed using SPSS 22.0 software. The Kolmogorov-Smirnov test was used to assess the normal distribution of continuous data, and the Mauchly ($p > 0.05$) and Levene tests ($p > 0.05$) were used to assess sphericity and homogeneity of variance, respectively. All data were normally distributed (Kolmogorov-Smirnov one-sample test: all $p > 0.05$). Demographic and clinical variables between groups were analysed using analysis of variance (ANOVA) for continuous variables and chi-square tests for categorical variables.

In this longitudinal study, the main aim was to examine the effects of four different intervention groups on depressive symptoms of MDD patients. Repeated measures (RM) multivariate analysis (MANOVA) was applied to analyze the change in HAMD scores from baseline to week four, with three main time points (baseline, week two,

week four) as within-effect repeated measures and four different intervention groups (A: active rTMS + active tDCS, B: active rTMS +sham tDCS, C: sham rTMS + active tDCS, D: sham rTMS +sham tDCS) as between-effect repeated measures. If the group × time interaction was significant, then the group difference at week 2 and 2-week follow-up was respectively analyzed by analysis of covariance (ANCOVA) with the baseline score as a covariate. If the interaction was not significant, no further statistical analyses were performed. To controlling for multiple testing, Bonferroni correction was applied.

We calculated changes in the HDRS-24 total score for the four groups (week two and week four minus baseline, respectively). Then we used ANOVA to compare the mean reduction in HDRS-24 total score between the four groups. Patients were in clinical remission if the HDRS-24 total score decreased to less than 9 points, whereas patients were considered to have a clinical response if the HDRS-24 total score decreased by 50%. Differences in the proportion of patients with clinical remission and response in each group were compared using the χ^2 test.
