# Supplementary material for: Transcranial Direct Current Stimulation Combined With Repetitive Transcranial Magnetic Stimulation for Depression: A Randomized Clinical Trial
Source: JAMA Netw Open. 2024 Nov 13;7(11):e2444306. doi: 10.1001/jamanetworkopen.2024.44306 (PMC11561687; doi:10.1001/jamanetworkopen.2024.44306)
Supplement: Supplement 2. — eMethods. Randomization, rTMS or tDCS Treatment and Blinding eResults. Dropout and Integrity of Blinding eTable 1. Odds Ratios (OR) and Risk Difference (RD) of Remission and Response Rates Between Groups eTable 2. Adverse Events Among Patients Receiving Treatment [file jamanetwopen-e2444306-s002.pdf]

Supplemental Online Content

Zhou D, Li X, Wei S, et al. Repetitive transcranial magnetic stimulation combined with transcranial direct current stimulation for depression: a randomized clinical trial. *JAMA Netw Open*. 2024;7(11):e2444306. doi:10.1001/jamanetworkopen.2024.44306

eMethods. Randomization, rTMS or tDCS Treatment and Blinding

eResults. Dropout and Integrity of Blinding

eTable 1. Odds Ratios (OR) and Risk Difference (RD) of Remission and Response Rates Between Groups

eTable 2. Adverse Events Among Patients Receiving Treatment

This supplemental material has been provided by the authors to give readers additional information about their work.

eMethods. Randomization, rTMS or tDCS Treatment and Blinding

Specifically, we used a computer-generated table of random numbers to create randomization sequence. All patients in the four groups (Group A: active tDCS + active rTMS, Group B: sham tDCS + active rTMS, Group C: active tDCS + sham rTMS, Group D: sham tDCS + sham rTMS) were randomly assigned at a ratio of 1:1:1:1 to ensure that each participant had an equal chance of being assigned to any group. Allocation concealment was implemented to prevent selection bias. The randomization sequence was generated by an independent researcher who was not involved in the clinical trial, and allocation information was managed using sealed, opaque envelopes.

These envelopes were sequentially numbered and contained group assignment information for each participant, which was only revealed by the staff member responsible for randomization once the participant had been formally enrolled in the study. All the equipment was of the same shape and color, each machine was given a number and each patient was given a fixed number. In addition, two sets of equipment were kept in case they were damaged and could not be used.

Throughout the study, the treatment procedures, the appearance of medications or epuipment, and the method of use were consistent across all groups to prevent unblinding. Both participants and researchers directly involved in treatment and assessment were blinded to group assignment. Operators used the appropriate equipment for each intervention according to the patient's number. Both researchers (operators, and raters) and patients were blinded to group assignment. We conducted two unblinding tests at the end of the clinical trial, both by professionals who retained a background in blinding. For the first unblinding, only the group to which each case belonged s was listed (e.g., Group 1, Group 2, Group 3, or Group 4). Statistical analyses were then performed, which were followed by a second unblinding to determine which active and control groups corresponded to Groups 1, 2, 3, or 4, respectively. In addition, we asked participants and raters separately whether they were aware of the true stimulus situation.

© 2024 Zhou D et al. *JAMA Network Open*

eResults. Dropout and Integrity of Blinding

In the active tDCS + active rTMS group one patient withdrew from treatment due to school exams and two feeling that treatment is ineffective. In the active rTMS group, four withdrew without reason, and one withdrew because he felt the treatment was ineffective. In the active tDCS group, three patients developed slight skin redness after receiving treatment and did not want to continue treatment. Another one changed medication during treatment and withdrew from treatment, and two other patients were discharged for administrative reasons. In the sham group, five withdrew because they felt the treatment was ineffective. two patients withdrew due to health problems that required a visit to comprehensive hospital. No patient dropped out due to serious side effects.

After receiving the intervention, 92.98% (53/57) of patient in Group A $_{\text{active tDCS + active rTMS}}$ thought that they had received the actual stimulation. In Group B $_{\text{sham tDCS + active rTMS}}$, 94.55% (52/55) of patients felt they had received actual stimulation. In Group C $_{\text{active tDCS + sham rTMS}}$, 98.15% (53/54) of patients felt that they had received stimulation. In Group D $_{\text{sham tDCS + sham rTMS}}$, as many as 84.91% (45/53) felt that they had received actual stimulation and that their feedback was significantly improved compared to before treatment.

Moreover, the ability of raters to accurately identify the treatment received by patients was tested. They correctly identified 34 out of 57 patients as having received both active stimulations, 33 out of 55 as having received sham tDCS and active rTMS, 24 out of 54 as having received sham rTMS with active tDCS, and 23 out of 53 as having been subjected to both sham stimulations in the sham tDCS + sham rTMS group. Collectively, these results indicate that neither patients nor raters could reliably discern the specific treatment modalities, affirming the effectiveness of the blinding process.

© 2024 Zhou D et al. *JAMA Network Open*

eTable 1. Odds Ratios (OR) and Risk Difference (RD) of Remission and Response Rates Between Groups

	2W		4w	
	remission	response	remission	response
OR				
Group A *vs* B	1.14	2.06	3.11	1.45
Group A *vs* C	3.00	13.25	1.98	1.22
Group A *vs* D	6.50	12.24	4.09	1.00
Group B *vs* C	2.62	6.40	0.64	0.84
Group B *vs* D	5.69	5.93	1.32	0.69
Group C *vs* D	2.17	0.92	2.07	0.82
RD				
Group A *vs* B	0.03	0.12	0.22	0.03
Group A *vs* C	0.25	0.55	0.11	0.02
Group A *vs* D	0.37	0.53	0.28	0.00
Group B *vs* C	0.22	0.43	-0.10	-0.02
Group B *vs* D	0.33	0.41	0.07	-0.03
Group C *vs* D	0.12	-0.02	0.17	-0.02

Abbreviation: rTMS = repetitive transcranial magnetic stimulation；tDCS = transcranial direct current stimulation；Group A: active tDCS + active rTMS; Group B: sham tDCS + active rTMS; Group C: active tDCS + sham rTMS; Group D: sham tDCS + sham rTMS.

© 2024 Zhou D et al. *JAMA Network Open*

eTable 2. Adverse Events Among Patients Receiving Treatment

Characteristic	Active tDCS + Active rTMS (N = 60)	Sham tDCS + Active rTMS (N = 60)	Active tDCS + Sham rTMS (N = 60)	Sham tDCS + Sham rTMS (N = 60)
Skin redness	3 (5)	0 (0)	3 (5)	1 (1.67)
Headache	3 (5)	3 (5)	0 (0)	2 (3.33)
Insomnia	0 (0)	2 (3.33)	0 (0)	2 (3.33)
Mild irritation	1 (1.67)	0 (0)	3 (5)	1 (1.67)
Pruritus	3 (5)	2 (3.33)	4 (6.67)	2 (3.33)
Dizziness	1 (1.67)	0 (0)	1 (1.67)	0 (0)
Nausea	0 (0)	1 (1.67)	0 (0)	0 (0)

Abbreviation: rTMS = repetitive transcranial magnetic stimulation；tDCS = transcranial direct current stimulation；

© 2024 Zhou D et al. *JAMA Network Open*
