# Supplementary material for: Transcranial Direct Current Stimulation Combined With Repetitive Transcranial Magnetic Stimulation for Depression: A Randomized Clinical Trial
Source: JAMA Netw Open. 2024 Nov 13;7(11):e2444306. doi: 10.1001/jamanetworkopen.2024.44306 (PMC11561687; doi:10.1001/jamanetworkopen.2024.44306)
Supplement: Supplement 3. — Data Sharing Statement [file jamanetwopen-e2444306-s003.pdf]

Data Sharing Statement

Zhou. Transcranial Direct Current Stimulation Combined With Repetitive Transcranial Magnetic Stimulation for Depression. *JAMA Netw Open*. Published November 13, 2024.
doi:10.1001/jamanetworkopen.2024.44306

Data
Additional Information: China Clinical Trial Registry (ChiCTR2100052122) / http://www.chictr.org.cn/showproj.aspx?proj=134520';.
Data available: Yes
Data types: Other (please specify)
Additional Information: apply to the author if there is a need for relevant data
How to access data: xingli0715@163.com
When available: With publication

Supporting Documents
Document types: Other (please specify)
Additional Information: Relevant data involved in the manuscript table
How to access documents: xingli0715@163.com
When available: With publication

Additional Information
Who can access the data: anyone requesting the data
Types of analyses: Overview or meta-analysis
Mechanisms of data availability: with investigator support
Any additional restrictions: no
